# Association of circulating microRNAs with prevalent and incident knee osteoarthritis in women: the OFELY study

**DOI:** 10.1186/s13075-019-2086-5

**Published:** 2020-01-02

**Authors:** Jean-Charles Rousseau, Marjorie Millet, Martine Croset, Elisabeth Sornay-Rendu, Olivier Borel, Roland Chapurlat

**Affiliations:** 1 INSERM 1033, Pavillon F, Hôpital E. Herriot, 69437 Lyon Cedex 03, France; 2 Hôpital E. Herriot, Hospices Civils de Lyon, Lyon, France; 3 Université de Lyon, Lyon, France

**Keywords:** Prevalent knee OA, Incident knee OA, Circulating miRNAs

## Abstract

**Objectives:**

In the context of the scarcity of biomarkers for knee osteoarthritis (OA), we examined the associations of prevalent and incident OA with the expression levels of serum miRNAs in subjects with and without OA.

**Methods:**

With a next-generation sequencing approach, we compared the miRome expression of 10 women with knee OA and 10 age-matched healthy subjects. By real-time qPCR, we analyzed the expression levels of 19 miRNAs at baseline selecting 43 women with prevalent knee OA (Kellgren Lawrence score of 2/3), 23 women with incident knee OA over a 4-year follow-up and 67 healthy subjects without prevalent or incident OA matched for age and body mass index.

**Results:**

Serum miR-146a-5p was significantly increased in the group of prevalent knee OA compared with controls (relative quantification (RQ); median [Interquartile range] 1.12 [0.73; 1.46] vs 0.85 [0.62; 1.03], *p* = 0.015). The likelihood of prevalent knee OA was significantly increased (odds ratio [95% confidence interval (CI)] 1.83 [1.21–2.77], *p* = 0.004) for each quartile increase in serum miR-146a-5p. The women with miR-146a-5p levels above the median (0.851) had a higher risk of prevalent knee OA compared to those below the median [95% CI] 4.62 [1.85–11.5], *p* = 0.001. Moreover, we found a significant association between the baseline level of serum miR-186-5p and the risk of incident knee OA (Q4 vs Q1–3; odds ratio [95% CI] 6.13 [1.14–32.9], *p* = 0.034).

**Conclusion:**

We showed for the first time that miR-146a-5p and miR-186-5p are significantly associated with prevalent and incident knee OA, respectively.

## Key messages

1. Circulating miR-146a-5p is significantly associated with prevalent knee OA in women.
2. Circulating miR-186-5p is significantly associated with incident knee OA in women.
3. Circulating miR-186-5p is a potential non-invasive biomarker of early-stage OA in women.

## Introduction

Osteoarthritis (OA) is the most frequent chronic musculoskeletal disease affecting approximately 40% of adults aged 70 years and over [1]. It is considered as a slowly progressive disease degrading all tissues of the affected joint, appearing as a destruction of the cartilage, the hallmark of OA, but also a mild-to-moderate synovial inflammation and an alteration of the subchondral bone structure. So far, the gold standard to estimate the extent of the disease has been plain radiography, but the poor sensitivity and the relatively large precision error lead to disease detection only when significant cartilage degradation has already occurred. Measuring circulating biomarkers appears as a potential non-invasive approach to diagnose the disease and prognosticate its evolution. Research has focused on the structural components of the cartilage matrix, especially type II collagen degradation markers [2]. Because of the large overlap between biomarker concentrations in controls and OA patients, the diagnosis of OA in an individual patient cannot be made by currently available biomarkers. Given the limitations of the tools that are currently available for OA assessment, there is considerable interest in the identification of specific biological markers that reflect quantitative and dynamic variations in joint tissue remodeling. For this purpose, microRNAs (miRNAs) are new potential targets. These small non-coding RNAs of 22–28 nucleotides in length can silence gene expression by binding to target messenger RNA (mRNA) repressing the translation. This regulation process is redundant because 1 miRNA can inhibit a large number of mRNA, and 1 gene can be targeted by multiple miRNAs. The miRNAs are important players in maintaining the health of all joint tissues including the cartilage, ligament, tendon, and muscle. Some of them have a potential role in regulating homeostasis of joint tissues in the context of OA [3]. Several in vitro and in vivo studies have reported the miRNA involvement in the OA onset and progression, by targeting cartilage-associated genes [4]. They regulate the expression of genes involved in pathways altered in OA chondrocytes such as apoptosis [5], expression levels of MMPs and ADAMTS [6–8], and chondrocyte signaling [9, 10]. To date, it has been reported that about 80 miRNAs are involved in the pathology of OA and that their circulating level in patients might reflect the underlying disease state [4]. Indeed, circulating miRNAs are considered to be attractive molecules to serve as biomarkers because they are easily accessible, stable in biofluids, and quantifiable with a high degree of sensitivity and specificity. Circulating miRNAs are protected from degradation because they circulate bound to proteins such as Argonaute 2 and nucleoplasmin or inside microvesicles such as exosomes [11–13]. Finally, although the expression of miRNAs has been well documented in OA cartilage, it remains difficult to understand to which extent circulating miRNAs reflect the tissue alteration. Moreover, the aberrant expression levels previously reported in circulating miRNAs differ considerably between clinical studies due to the different characteristics of these settings [14–22].

In this study, a first screening phase was conducted by comparing the serum miRome in subjects with (*n* = 10) and without (*n* = 10) knee OA in the OFELY cohort by next-generation sequencing (NGS) analysis. Then, on the basis of our NGS data and on the previously reported function of miRNAs in the OA process, both in basic and/or clinical research, we have selected 19 candidate miRNAs for quantitative real-time polymerase chain reaction (RT-qPCR) analysis. By conducting the validation phase of these candidate miRNAs in the OFELY cohort, we report that miR-146a-5p and miR-186-5p are significantly associated with prevalent and incident knee OA, respectively.

## Methods

### Patients selected from the OFELY cohort

The study group for both steps, NGS and RT-qPCR, included French women belonging to a population-based cohort. These women were participants in a prospective investigation of the determinants of bone loss, the Os des FEmmes de LYon (OFELY) study. This cohort has previously been described in detail elsewhere [23, 24]. In the present analysis, we studied the miRNA expression in women with knee OA at baseline for this analysis, 8 years after the recruitment of the cohort (ninth follow-up visit). The primary or post-traumatic etiology of knee OA was not known, but the selected women had no other skeletal diseases. The evaluation of OA disease was performed by radiography for knee OA and spine disc degeneration, by clinical examination for hand OA, and by a questionnaire for the hip OA (for details on the OA evaluation, see [8]). After baseline OA assessment, women were followed prospectively for 4 years.

### Assessment of knee OA

Radiographs of both knees were obtained in all women. Radiologic evaluation of the knees consisted of bilateral posteroanterior weight-bearing knee radiographs with fixed flexion using the SynaFlex X-ray Positioning Frame (Synarc, San Francisco, CA), as previously described [25]. Radiographs were obtained in a single radiography unit by the same staff of two technicians using a previously described standardized protocol [26]. The severity of OA was performed and graded according to Kellgren and Lawrence (KL) classification at baseline and 4 years later [27]. Prevalent knee OA was defined by a KL score higher or equal to 2 at baseline. Incident OA was defined by a KL score higher or equal to 2 at year 4 and a KL score < 2 at baseline. All knee radiographs were scored by a single trained rheumatologist (ES-R). Measurements were made paired but not blinded to sequence, which has been shown not to modify the sensitivity to change [28].

### The miRNA analysis by next-generation sequencing

#### Selection of patients

**Fig. 1:**
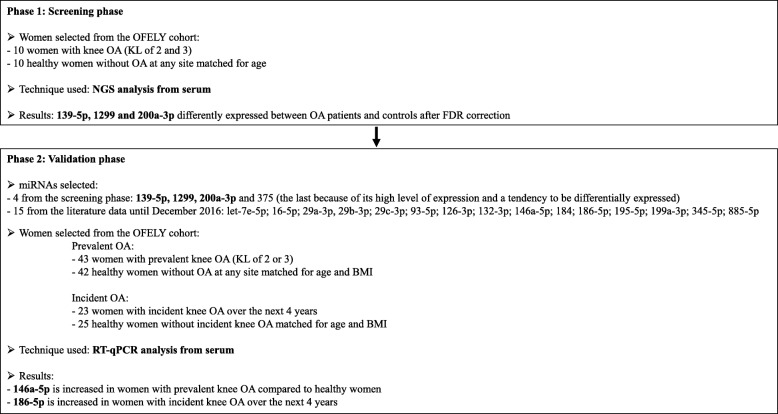
The flow chart of the study

The expression levels of miRNAs in the serum were measured in ten women with knee OA (KL score of 2 and 3) and in ten healthy women without OA at any site (for details, see [24]). Both groups were matched for age (healthy 61.9 ± 3.03 years and OA 63.9 ± 3.4 years, *p* = 0.17). The flow chart for the design of the experimental study is reported in Fig. 1.

#### NGS analysis

Total RNA was extracted from 400 μl of serum with the miRCURY Biofluids extraction kit (Exiqon®, Denmark) and analyzed by small RNA sequencing on a NextSeq500 sequencing instrument (Illumina platform). After RNA conversion into miRNA NGS libraries using the NEBNEXT library generation kit, cDNA was pre-amplified prior to library purification and quantification (for detailed procedures of extraction, library preparation and purification, normalization and quality controls, see Additional file 1: Data S1). Measurements were expressed as tags per million (TPM) in which the number of reads for a particular miRNA is divided by the total number of mapped reads in a sample and multiplied by 10^6^. The differential expression of miRNA between the OA and control groups was expressed by the TPM ratio converted as the Log_2_ (fold change) (Log_2_FC).

### The miRNA analysis by real-time quantitative polymerase chain reaction

#### Selection of patients

All women were postmenopausal and belonged to the OFELY cohort. The expression levels of selected miRNAs were measured, for the prevalent OA, in 43 women with prevalent knee OA (KL score of 2 and 3; early and intermediate knee OA, age 68.3 ± 6.6 years, body mass index (BMI) 26.6 ± 4.4 kg/m^2^) and in 42 healthy women without knee OA matched for age and BMI, and for the incident OA, in 23 women with incident knee OA over the next 4 years (age 68.4 ± 8 years, BMI 25.2 ± 4 kg/m^2^) and in 25 healthy subjects without incident knee OA matched for age and BMI (Table 1).

**Table 1:** Baseline characteristics of postmenopausal women included in the knee OA study with the localization of OA, if present at other sites than the knee

		Quantitative real-time PCR					
		Prevalent OA			Incident OA		
		Control	Knee OA	*p* value	Control	Knee OA	*p* value
Number of subjects		42	43		25	23	
Age (years, mean ± SD)		68.3 ± 6.6	68.3 ± 6.6	0.77	67.7 ± 7.2	68.4 ± 8.0	0.72
BMI (kg/m^2^)		25.9 ± 4.4	26.6 ± 4.4	0.38	26.2 ± 5.2	25.2 ± 4.0	0.73
Knee OA	KL score at baseline	0	2/3		0	0/1	
	KL score at the end of the follow-up	0/1	2/3/4		0	2/3	
OA at other sites	Hip (Lane score)	2	5	0.25	5	5	0.88
	Hand (ACR criteria)	23	25	0.75	11	7	0.33
	Spine (self-reported)	34	37	0.67	20	21	0.19

#### Preselection of miRNAs

The first selection of candidate miRNAs was based on the data obtained from the initial screening phase. We have selected miR-139-5p, miR-200a-3p, and miR-1299 in regard to their significant differential expression between the groups after Benjamini-Hochberg false discovery rate (FDR) correction. We have also added miR-375 for its high level of expression and a tendency to be differentially expressed, despite the absence of significant difference after FDR correction. Then, a second miRNA selection was performed by a critical assessment of literature data until December 2016 to highlight miRNAs that are differentially expressed in the biofluids of patients with knee OA vs controls. We have considered the criteria for patient selection, the size of the groups studied, the technical approaches used for screening and validation of miRNAs in biofluids, and the involvement of these differentially expressed miRNAs in the regulation of metabolic process contributing to OA pathology (see Table 2). Thus, we have further selected 15 candidate miRNAs: let-7e-5p, 16-5p, 29a-3p, 29b-3p, 29c-3p, 93-5p, 126-3p, 132-3p, 146a-5p, 184, 186-5p, 195-5p, 199a-3p, 345-5p, and 885-5p.

**Table 2:** A summary of studies on human miRNAs with dysregulated expression in patients suffering from OA, including the biological fluid tested, the number of patients with their baseline characteristics, the criteria for patient selection, the quantification methods, and the miRNAs significantly dysregulated

Murata K, 2010 [13]	Plasma and synovial fluid	Plasma: 30 knee OA, 30 RA, 30 CTL Synovial fluid: 30 OA, 30 RA	Plasma: OA: 75,1 yrs, 77 % ; RA: 60.1 yrs, 73%; CTL: 46,5 yrs, 57 % Synovial fluid: RA: 63,1, 80%; OA: 75,3, 80%	RA and knee OA were diagnosed according to the ACR criteria no indication of OA at other sites	
Zhang L, 2012 [14]	Serum	Screening: 5 OA, 5 RA and 6 CTL Validation: 102 ACL and 60 CTL	41 yrs or youngers	one year after anterior cruciate ligament injury no indication of OA at other sites	
Okuhara A, 2012 [15]	Peripheral blood mononuclear cells	36 OA 36 CTL	OA: 68 yrs, 81 % CTL: 32 yrs, 47 %	Knee OA ACR criteria no indication of OA at other sites	
Borgonio-Cuadra VM, 2014 [16]	Plasma	Screening: 14 OA and 5 CTL Validation: 27 OA and 27 CTL	Screening: OA: 55.7 yrs, 71.4 % CTL: 47.5 yrs, 100% Validation: OA: 55.6 yrs, 88.9% CTL: 52.9 yrs, 81.5%	Knee OA, KL 2/3 and BMI < 27 no indication of OA at other sites	
Beyer C, 2015 [17]	Serum	Screening: pooled serum from 13 individuals with knee/hip arthroplasty Pooled serum from 13 individuals without knee/hip arthroplasty Validation: 749 OA and 67 CTL	OA: 65 yrs, 58.2%CTL: 62.7 yrs, 49.3%	Knee/hip arthroplasty (KL 3,4) no indication of OA at other sites	
Li YH, 2016 [18]	Synovial fluid	Screening: 4 early OA and 4 late OA Validation: 22 early OA and 26 late OA	Screening: Early OA: 51 yrs, 100 % Late OA: 64 yrs, 100% Validation: Early OA: 56 yrs, 36.4% Late OA: 60 yrs, 61.5%	Early stage OA: degenerative meniscal tears undergoing arthroscopic surgery (KL grade 1,2) Late stage OA: total knee replacement surgery (KL grade 3,4) no indication of OA at other sites	
Soyocak A, 2017 [19]	Peripheral blood mononuclear cell	100 patients with knee OA and 50 CTL	OA: from 47 to 70yrs, 84% CTL: from 35 to 38 yrs, 84%	Knee OA, ACR criteria no indication of OA at other sites	
Kong R, 2017 [20]	Plasma	Screening: 8 knee OA and 8 CTL Validation: 100 OA and 100 CTL	Screening: OA: 51.13 yrs, 62.5% CTL: 50.75 yrs, 62.5% Validation: OA: 51.69 yrs, 69% CTL: 51.09 yrs, 61%	Knee OA, ACR criteria no indication of OA at other sites	
Ntoumou E, 2017 [21]	serum	Screening: 12 primary OA and 12 CTL Validation: 12 OA and 12 CTL	Screening: OA: 69,8 yrs, 75% CTL: 64,2 yrs, 50% Validation: not indicated	Knee OA, KL ≥3 no indication of OA at other sites	
Murata, 2010	High pure miRNA Isolation kit (Roche)	Ncode VILO miRNA cDNA Synthesis kit (Invitrogen)	Express SYBR GreeER qPCR Supermix (Invitrogen) control: cel-miR-39	Applied Biosystems 7300 SDS Relative Quantification 1.3 (Applied Biosystems)	Plasma miRNAs had distinct pattern from SF miRNAs miR-132: potential diagnostic marker for patients with OA or RA
Zhang, 2012	miRNeasy kit (Quiagen)	Validation: TaqMan miRNA reverse transcription kit (Invitrogen) + a pulsed RT reaction with a Eppendorf mastercycler (Eppendorf)	Screening: Megaplex RT human pool A and B (Applied) Validation: Preamp, TaqMan PreAmp master mix; qPCR, TaqMan qPCR assays control: U6 (Applied)	Screening: 7900HT (Applied) Validation: 7500 (Applied) SDS Relative Quantification 2.2.3 (Applied)	U38 and U48 upregulated in patients developing cartilage damage at one year after ACL injury
Okuhara, 2012	Trizol reagent (Invitrogen)	Thermocycler (BioRad)	TaqMan miRNA assay kit (Applied) control: U18	Mini Opticon Real-time PCR System (BioRad)	146, 155, 181a, 223 upregulated in OA vs CTL ealy stage: 146a and 223 higher than in late stage
Borgonio-Cuadra, 2014	Mini miRNeasy kit (Quiagen)	Screening: RT Megaplex Pool A on a GeneAmp PCR 9700 System (Applied) Validation: specific miRNA primer and TaqMan probes (Applied)	Screening: preamp with Megaplex PreAmp MasterMix, TLDA ver.2.0 plate A (Applied) Validation: : preamp with Megaplex PreAmp MasterMix, qPCR control: U6	Screening and Validation: : 7900HT (Applied)	12 miRNAs overexpressed in OA vs CTL: 16, 20b, 29c, 30b, 93, 126, 146a, 184, 186, 195, 345, 885-5p
Beyer, 2015	Mini miRNeasy kit (Quiagen)	Megaplex Primer Pools (Human Pools A V.2.1) (Applied)	Screening: Human TaqMan miRNA Array Card A V.2.1 (Applied) Validation: TaqMan miRNA assays (Applied) control: U6 or Ct average of all miRNA measurements for each sample	Screening and validation: 7900HT (Applied) SDS 2.2 software (Applied) and LinRegPCR software	let-7e, 454, 885-5p potential predictors for severe knee or hip OA
Li YH, 2016	miRCURY RNA isolation kit-biofluids (EXIQON)	Universal cDNA synthesis kit II (EXIQON)	miRNA ready-to-use PCR array (Human panel I + II, EXIQON) using ExiLENT SYBR Green master mix (EXIQON)	not indicated	23a-3p, 24-3p, 27b-3p, 29c-3p, 34a-5p, 186-5p upregulated and 27a-5p, 329, 655, 708-3p, 934 downregulated in late stage OA ve early OA 27a-3p, 101-5p, 378-5p only expressed in late stage
Soyocak A, 2017	miRVana miRNA Isolation kit (Applied)	TaqMan MicroRNA Reverse transcription Kit (Applied)	TaqMan Small RNA Assays, TaqMan Gene Expression Master Mix control: U44 and 18S	qPCR system (Mx3000p, Stratagene)	miR-155: increased in OA miR-146a and miR-155 increased in the progressive stages
Kong R, 2017	LeukoLOCK kit (Ambion)	Screening: microarray hybridation with the GeneChip miRNA 4.0 Array (Affymetrix) Validation: TaqMan microRNA Reverse Transcription kit (Life Technologies)	Validation: TaqMan miRNA assays (Applied) control: U6	7900HT (Applied)	19b-3p, 92a-3p, 122-5p, 486-5p, 320b increased in OA 19b-3p, 122-5p, 486-5p, great diagnostic value 19b-3p and 486-5p positively corretated with disease severity
Ntoumou E, 2017	RiboEXTMLS kit (Geneall)	Screening: miRNA complete labeling and hybridization kit (Agilent) using SurePrint G3 Human miRNA 8X60K platform (Agilent) Validation: miScript II Reverse Transcription Kit (QIAGEN)	Validation: quantification with miScript SYBR Green PCR kit and miScript Primer Assays (QIAGEN) Control: Hsa-miR-25-1	Screening: Agilent Feature Extraction Software version 4.0.1.21 Validation: ABI 7300 Real-time PCR system (Applied)	3 miRNAs significantly downregulated in OA patients: hsa-miR-140-3p, hsa-miR-33b-3p, hsa-miR-671-3p

*CTL* control, *OA* osteoarthritis, *yrs* years, *BMI* Body Mass Index, *KL* Kellgren et Lawrence, *ACR* American College of Rheumatology, *Preamp* preamplification

### RNA isolation and miRNA RT-qPCR analysis

Total RNA was extracted from 200 μl of serum (miRCURY RNA isolation kit for biofluids, EXIQON, Danemark) by adding a lysis solution containing cel-miR-39-3p as exogenous control, followed by protein precipitation and miRNA purification on a silica column. miRNAs quantification was performed by the TaqMan® Advanced miRNA technology (Applied Biosystems, Thermo Fisher Scientific) in which miRNA was reverse-transcribed and cDNA pre-amplified with the TaqMan Advanced miRNA-cDNA synthesis kit, followed by analysis on pre-designed miRNA TaqMan arrays (TLDA) according to the manufacturer’s protocols (Applied Biosystems, CA, USA). Amplified cDNAs (15 μl) were mixed with 75 μl of TaqMan Fast Advanced Mastermix and water (60 μl), and 100 μl of the sample was added to each array tank of the card. The TLDA wells were pre-spotted with TaqMan®Advanced miRNA assays that allow the quantification in duplicate of 19 miRNAs (Table 3) by RT-qPCR reaction on a QuantStudio7 flex (Applied Biosystems) according to the manufacturer’s protocol. The Ct values recorded by the software Expression Suite were normalized by the Ct mean of 3 endogenous miRNAs for the relative quantification (RQ) of miRNA levels as fold change (FC) = Log_2_(2^−ΔΔCT^) (for protocol details, see Additional file 1: Data S1).

**Table 3:** Identification of the miRNAs analyzed in the validation phase and of the endogenous and exogenous miRNA normalizers. Each miRNA is identified by its NCBI accession number and sequence, according to miRBase v20. The pre-designed TaqMan Advanced miRNA Assays used to quantify the serum miRNA level are specified by their identification number (Applied Biosystems, Thermo Fisher Scientific)

miR base ID	NCBI accession number	TaqMan™ Advanced miRNA Assay (ID)	Sequence of the mature miRNA (5′-3′)
hsa-miR-139-5p	MIMAT0000250	478312_mir	UCUACAGUGCACGUGUCUCCAGU
hsa-miR-200a-3p	MIMAT0000682	478490_mir	UAACACUGUCUGGUAACGAUGU
hsa-miR-1299	MIMAT0005887	478696_mir	UUCUGGAAUUCUGUGUGAGGGA
hsa-let-7e-5p	MIMAT0000066	478579_mir	UGAGGUAGGAGGUUGUAUAGUU
hsa-miR-16-5p	MIMAT0000069	477860_mir	UAGCAGCACGUAAAUAUUGGCG
hsa-miR-29a-3p	MIMAT0000086	478587_mir	UAGCACCAUCUGAAAUCGGUUA
hsa-miR-29b-3p	MIMAT0000100	478369_mir	UAGCACCAUUUGAAAUCAGUGUU
hsa-miR-29c-3p	MIMAT0000681	479229_mir	UAGCACCAUUUGAAAUCGGUUA
hsa-miR-93-5p	MIMAT0000093	478210_mir	CAAAGUGCUGUUCGUGCAGGUAG
hsa-miR-126-3p	MIMAT0000445	477887_mir	UCGUACCGUGAGUAAUAAUGCG
hsa-miR-132-3p	MIMAT0000426	477900_mir	UAACAGUCUACAGCCAUGGUCG
hsa-miR-146a-5p	MIMAT0000449	478399_mir	UGAGAACUGAAUUCCAUGGGUU
hsa-miR-184	MIMAT0000454	477938_mir	UGGACGGAGAACUGAUAAGGGU
hsa-miR-186-5p	MIMAT0000456	477940_mir	CAAAGAAUUCUCCUUUUGGGCU
hsa-miR-195-5p	MIMAT0000461	477957_mir	UAGCAGCACAGAAAUAUUGGC
hsa-miR-199a-5p	MIMAT0000231	478231_mir	CCCAGUGUUCAGACUACCUGUUC
hsa-miR-345-5p	MIMAT0000772	478366_mir	GCUGACUCCUAGUCCAGGGCUC
hsa-miR-375	MIMAT0000728	478074_mir	UUUGUUCGUUCGGCUCGCGUGA
hsa-miR-885-5p	MIMAT0004947	478207_mir	UCCAUUACACUACCCUGCCUCU
hsa-miR-191-5p	MIMAT0000440	477952_mir	CAACGGAAUCCCAAAAGCAGCUG
hsa-miR-222-3p	MIMAT0000279	477982_mir	AGCUACAUCUGGCUACUGGGU
hsa-miR-361-5p	MI0000760	481127_mir	UUAUCAGAAUCUCCAGGGGUAC
cel-miR-39-3p	MI0000010	478293_mir	UCACCGGGUGUAAAUCAGCUUG

### Statistical analysis

A miRNA with a *p* value < 0.05 and a false discovery rate of 5% [Benjamini-Hochberg false discovery rate (FDR) correction for NGS approach] were considered as differentially expressed. Wilcoxon tests were used to compare the miRNA levels between women with and without OA because of the skewness of the data. We have examined the likelihood of OA, expressed as odds ratios (ORs) and 95% confidence intervals (CIs), per quartile increase in miRNA levels in a logistic regression model. All statistical analyses were performed using Stata 12 (Stata Corp LP, College Station, TX, USA).

## Results

### Screening: serum miRNA profiling of patients with knee OA and controls

We identified 421 miRNAs with an expression level ≥ 1 TPM and 241 with an expression level ≥ 10 TPM. When we compared both groups, 22 miRNAs showed differential expression (*p* < 0.05) between controls and OA patients, 13 upregulated and 9 downregulated. After the Benjamini-Hochberg FDR correction, hsa-miR-139-5p, hsa-miR-1299, and hsa-miR-200a-3p remained significantly differently expressed between OA patients and controls (*p* < 0.05, FDR at 5%) (Table 4).

**Table 4:** Differential expression of the miRNA level in the serum from controls and OA patients, analyzed by next-generation sequencing

Names	Log2 fold change	*p* value	FDR adjusted *p* value	Healthy average TPM	OA average TPM
hsa-miR-139-5p	0.73	0.0001	0.0434	90.1	143.3
hsa-miR-1299	− 3.38	0.0002	0.0434	12	0.8
hsa-miR-200a-3p	− 1.88	0.0003	0.0473	77.2	29.4
hsa-miR-129-5p	0.81	0.0022	0.1640	3.6	6.5
hsa-miR-429	− 1.43	0.0023	0.1640	5.5	2.5
hsa-miR-9-5p	0.83	0.0045	0.2410	3.2	6.1
hsa-miR-375	− 0.93	0.0065	0.2795	737	411.3
hsa-miR-139-3p	0.61	0.0072	0.2820	48.8	69.7
hsa-miR-200b-3p	− 1.18	0.0089	0.3193	28	15.7
hsa-miR-150-5p	0.72	0.0164	0.4619	173.6	252.6
hsa-miR-192-5p	− 1.03	0.0171	0.4619	1837.8	1161.9
hsa-miR-155-5p	0.52	0.0193	0.4907	64.9	87.5
hsa-miR-15b-3p	− 0.73	0.0247	0.5867	43.8	24.3
hsa-miR-1228-5p	0.78	0.0295	0.5918	4.9	8.7
hsa-miR-126-3p	0.38	0.0304	0.5918	3243.9	3976.7
hsa-miR-206	1.10	0.0338	0.6011	51.3	95.9
hsa-miR-199b-5p	− 0.57	0.0373	0.6011	29.9	18.6
hsa-miR-1246	0.67	0.0375	0.6011	29.9	46.9
hsa-miR-10b-5p	0.46	0.0428	0.6390	3848.4	5039.8
hsa-miR-642a-3p	0.69	0.0444	0.6405	4.5	7.2
hsa-miR-186-5p	− 0.45	0.0473	0.6405	777.7	534
hsa-miR-10a-5p	0.45	0.0493	0.6469	2613.1	3361.1

When we compared the OA vs healthy groups, 22 miRNAs showed differential expression (*p* < 0.05) between controls and OA patients. After Benjamini-Hochberg false discovery rate (FDR) correction, hsa-miR-139-5p, hsa-miR-1299, and hsa-miR-200a-3p remained significantly different between OA patients and controls (*p* < 0.05, FDR at 5%)

### Validation: differential expression of candidate miRNAs in serum of patients with knee OA and controls

#### Prevalent knee OA and miRNA-146a-5p

**Fig. 2:**
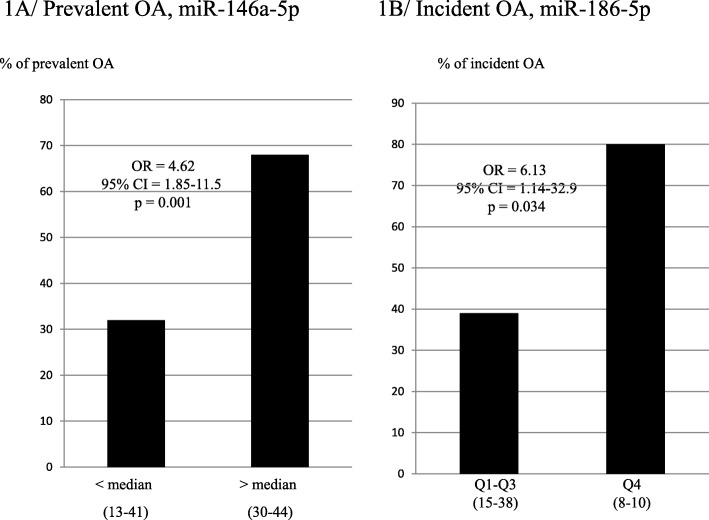
Risk of prevalent and incident OA according to the median of miR-146a-5p and the quartiles of miR-186-5p, respectively. Multiple logistic regression analyses to determine the ability of serum miR-146a-5p and miR-186-5p levels to predict prevalent or incident OA, respectively. **a** Prevalent OA: percentage of women with osteoarthritis under and upper the median of miR-146a-5p (number of women with prevalent OA − total number of women). **b** Incident OA: percentage of women with osteoarthritis in the first three quartiles vs the upper quartile (number of women with incident OA − total number of women). CI, confidence interval; OD, odds ratio

When considered as a continuous variable, miR-146a-5p was significantly increased in the group of prevalent knee OA compared with controls (relative quantification (RQ); median [interquartile range] 1.12 [0.73; 1.46] vs 0.85 [0.62; 1.03], *p* = 0.015). The likelihood of prevalent knee OA was significantly increased (odds ratio [95% confidence interval (CI)] 1.83 [1.21–2.77], *p* = 0.004) for each quartile increase in serum miR-146a-5p. Moreover, the women with miR-146a-5p levels above the median (0.851) had a greater risk of prevalent knee OA compared to those below the median [95% CI] 4.62 [1.85–11.5], *p* = 0.001 (Table 5 and Fig. 2a and Additional file 2: Data S2 for the 19 miRs tested, mean and standard deviation (SD) for each quartile).

**Table 5:** Differential expression of the miRNA level in the serum from controls and OA patients analyzed by quantitative real-time PCR

miR	Prevalent OA (−), median (IQR 25–75%)	Prevalent OA (+), median (IQR 25–75%)	*p* value	Incident OA (−), median (IQR 25–75%)	Incident OA (+), median (IQR 25–75%)	*p* value
126-3p	1.08 (0.85–1.36)	1.06 (0.89–1.30)	0.75	0.89 (0.80–1.04)	0.91 (0.73–1.23)	0.66
1299	0.28 (0.16–1.73)	0.33 (0.16–2.35)	0.80	0.27 (0.14–11.36)	0.34 (0.12–0.90)	0.82
132-3p	1.37 (0.71–2.12)	0.86 (0.43–1.85)	0.12	0.91 (0.63–1.19)	1.04 (0.59–1.97)	0.70
139-5p	1.50 (0.34–3.63)	2.09 (0.23–8.24)	0.27	0.28 (0.05–1.98)	0.81 (0.14–6.59)	0.16
146a-5p	0.85 (0.62–1.03)	1.12 (0.73–1.46)	0.015	0.83 (0.70–0.97)	0.88 (0.62–1.29)	0.94
16-5p	0.92 (0.72–1.82)	0.85 (0.61–1.50)	0.35	0.98 (0.59–1.24)	1.01 (0.73–1.44)	0.33
184	0.95 (0.64–2.18)	1.05 (0.58–2.38)	0.86	0.69 (0.41–1.76)	0.80 (0.31–1.24)	0.89
186-5p	1.09 (0.81–1.39)	1.03 (0.79–1.31)	0.47	0.82 (0.72–1.09)	1.05 (0.82–1.46)	0.09
195-5p	0.91 (0.75–1.42)	0.90 (0.52–1.60)	0.50	0.93 (0.65–1.24)	1.03 (0.69–1.47)	0.42
199a-3p	0.96 (0.77–1.12)	0.93 (0.82–1.47)	0.46	0.89 (0.76–0.98)	1.00 (0.71–1.39)	0.13
200a-3p	0.84 (0.56–1.93)	1.00 (0.58–1.57)	0.62	0.72 (0.49–1.06)	0.73 (0.48–1.43)	0.93
29a-3p	1.12 (0.76–1.44)	1.00 (0.68–1.38)	0.40	1.12 (0.63–1.35)	0.84 (0.61–1.50)	0.66
29b-3p	1.14 (0.81–1.74)	1.06 (0.81–1.51)	0.38	1.02 (0.71–1.42)	0.99 (0.67–1.53)	0.93
29c-3p	1.04 (0.66–1.59)	0.91 (0.64–1.59)	0.63	0.73 (0.44–1.16)	0.86 (0.45–1.56)	0.35
345-5p	3.10 (0.10–10.01)	2.81 (0.22–7.12)	0.99	0.53 (0.05–2.11)	1.14 (0.09–5.97)	0.24
375	1.57 (0.20–4.05)	1.40 (0.48–4.77)	0.56	0.98 (0.26–2.99)	0.86 (0.24–3.07)	0.91
885-5p	1.40 (0.60–2.16)	1.16 (0.28–2.40)	0.48	0.97 (0.49–2.85)	1.54 (0.70–2.38)	0.54
93-5p	1.11 (0.79–1.51)	0.91 (0.68–1.38)	0.21	1.07 (0.79–1.17)	1.19 (0.79–1.53)	0.25
let-7e-5p	1.15 (0.41–4.52)	2.28 (0.64–5.83)	0.25	1.36(0.78–2.72)	1.37 (0.36–2.36)	0.89

The miRNA levels were analyzed in the serum from individuals of the validation cohort, and differences in the serum miRNA levels are reported according to the prevalent or incident OA status. The relative miRNA level was used for the statistical comparisons. IQR = interquartile range [(+) = women with prevalent or incident knee OA, (−) = controls]

#### Incident knee OA and miRNA-186-5p

When considered as a continuous variable, serum miR-186-5p levels were not significantly associated with an increased risk of incident knee OA (*p* = 0.09). However, in an analysis across quartiles, we found a significant association between baseline miR-186-5p levels and the likelihood of incident knee OA for each quartile increase (odds ratio [95% CI] 1.71 [1.00–2.95], *p* = 0.049). Women in the upper quartile were 6.13 times more likely to develop incident knee OA over the next 4 years compared to those in the three others quartiles (Q4 vs Q1–3; odds ratio [95% CI] 6.13 [1.14–32.9], *p* = 0.034) (Table 5 and Fig. 2b).

## Discussion

In the present work, we have studied the serum miRNA profiling of women with knee OA to identify potential non-invasive biomarkers of the disease. We first screened the serum miRNAs with significant differences in the expression between women with knee OA and healthy controls by a NGS approach. Then, we have conducted a validation study in the OFELY cohort of women having an incident or prevalent knee OA with a long-time follow-up. In this, we analyzed by RT-qPCR whether the 3 differentially expressed miRNAs (139-5p, 200a-3p, 1299) in patients with knee OA in the screening step were also aberrantly expressed in the women with incident and prevalent knee OA of the validation cohort. In addition, miR-375 has been chosen because of its high expression level and a tendency to be differentially expressed in our NGS approach despite the absence of significant difference after FDR correction. Moreover, we have measured the expression levels of 15 additional miRNAs previously highlighted as having a dysregulated expression in patients with knee OA*.* The miRs-29 family (a, b, c) and miR-199a-3p were chosen for their activity as key regulators of chondrocyte gene expression with aberrant expression in OA cartilage [29–31]. The miRNAs let-7e-5p, 16-5p, 93-5p, 126-3p, 132-3p, 146a-5p, 184, 186-5p, 195-5p, 345-5p, and 885-5p were selected because previous studies analyzing circulating miRNAs in the serum and plasma [14, 17, 18] have reported their dysregulated expression in patients with knee OA (see Table 4).

The NGS approach revealed that miR-139-5p, miR-1299, and miR-200a-3p had levels of expression significantly different between OA patients and controls. However, we did not validate these 3 miRNAs in the second step of the analysis when we measured their level in the largest validation cohort. This lack of validation might come from the small number of samples in the discovery step (10 OA vs 10 non-OA) even if this number is comparable to those used in previous studies (Beyer: 13 OA vs 13 non-OA [18]; Borgonio-Cuadra: 14 OA vs 5 non-OA [17]; Kong: 8 OA vs 8 non-OA [21]) and/or differences in the specificity and sensibility of the platforms used (Illumina for this study; Borgonio-Cuadra [17] and Beyer [18]: Applied Biosystems; Kong [21]: Affimetrix).

It appears that the previous studies performed with biological fluids are difficult to compare each other because of the heterogeneity of the clinical situations (see Table 2). Nevertheless, our study can be compared to that of Borgonio-Cuadra et al. [17], particularly the patient selection is based on the same criteria, radiologic KL scores of 2 and 3 and a BMI smaller than 27. However, among the ten miRNAs in common, only miR-146a-5p (miR-146) showed significant overexpression, emphasizing the challenges faced with patient heterogeneity. The reasons for this discrepancy could be the ethnic origin, Mexican vs European, enhancing the differences in exposure to environmental factors; the sex of the participants, men and women vs women only; the biological fluid used-plasma vs serum; or the mean age of participants, 55 vs 68 years.

Nevertheless, we found that miR-146a-5p expression is significantly increased in the OA group of postmenopausal women compared to healthy controls independently of age and BMI. Moreover, we reported for the first time that the risk of prevalent knee OA was significantly increased for each quartile increase in the serum level of miR-146a-5p. However, the interpretation of this increase remains challenging in regard to the dual role of miR-146a-5p in the pathophysiology of osteoarthritis. MiR-146 is expressed in chondrocytes which begin to undergo degenerative changes [32]; its expression is stimulated by pro-inflammatory cytokines in an NF-κB-dependent manner [33]. It may contribute to OA by impairing the TGF-β signaling pathway targeting Smad4 and increasing apoptosis [5, 34]. Recently, Zhang et al. showed that miR-146 facilitates OA progression by targeting Camk2d and Ppp3r2 that are required to maintain the phenotype of mature chondrocytes [33].

In contrast, miR-146 could play a protective role in OA. It is induced during hypoxia by HIF-1 and promotes autophagy by decreasing the Bcl-2 expression, an autophagy inhibitor. Autophagy has a protective function for cartilage during OA [35]. Moreover, miR-146 exerts negative control on inflammatory responses by suppressing IRAK1 and TRAF6, two molecular targets of miR-146 impairing NF-kβ activity and suppressing NF-kβ target genes such as IL-6 and TNF-α [36, 37]. Recently, Zhong et al. [38] showed that miR-146 could increase the proliferation and inhibit apoptosis of OA chondrocytes by inhibiting TRAF6 expression and suppressing the activation of the NF-κB signaling pathway. Finally, Guan et al. [39] showed that miR-146 is activated by cyclic loads in the physiological range but suppressed by mechanical overload in human articular chondrocytes. They proposed that miR-146 protects joint cartilage from degeneration through inhibition of the feedback loop of Notch-1/IL-6 during aging or trauma.

It must be noted that circulating miR-146 has been highlighted in two other studies analyzing the peripheral blood mononuclear cells. Okuhara et al. [16] found it overexpressed in patients suffering from knee OA compared to healthy controls and highly expressed in OA peripheral blood mononuclear cells from patients with early OA. Soyocak et al. [20] observed that, in patients classified according to the KL score, miR146a expression increased in the progressive stages (grades 3 and 4).

Finally, miR-146 is one of the four miRNAs with miR-223, miR-16, and miR-30b that were differently expressed in the cartilage and in the circulation with a role in the cartilage homeostasis [4]. Moreover, miR-146 is also involved in the inhibition of osteoclastogenesis [40] suggesting that its measurement in the serum could reflect the deregulations affecting the cartilage and bone tissues. Although the activity of this miRNA seems dependent on the disease phases, it appears as a key element in the OA pathophysiology, and our results suggest that miR-146a-5p could be a new biological marker for knee OA.

We report for the first time a significant association, independent of age and BMI, of serum miR-186-5p and incident knee OA risk in a population of postmenopausal women followed prospectively for 4 years. This result is in accordance with Borgonio-Cuadra et al. [17] showing that miR-186-5p was overexpressed in the plasma of early-stage OA patients compared to controls. In silico analysis has suggested that potential pathways regulated by miR-186 could be signaling by PDGF, developmental biology, membrane trafficking, and collagen formation. However, Li et al. [19] showed that miR-186 originated from the synovial membrane and belongs to a panel of seven miRNAs that were significantly upregulated in late-stage OA synovial fluid compared to early-stage irrespective of age, gender, and BMI. The reasons for this discrepancy are not clear but could be the geographic origin of patients, North America vs Europe, or the biological fluids analyzed, synovial fluid vs serum/plasma. Moreover, patients from Li’s study were selected from a group with degenerative meniscal tears undergoing arthroscopic surgery (early OA) or total knee replacement (late-stage OA) while Borgonio-Cuadra et al. [17] and us have selected patients on the radiographic score only, without any consideration regarding the OA etiology.

Together with the serum association of miR-146a-5p and prevalent OA, these data reinforce the notion that the miRNAs involved in OA pathophysiology vary according to the stage of the disease [19]. Finally, miR-186 has been essentially studied in cancer, Alzheimer’s disease, neuropathic disorder, and acute myocardial infarction [41]. It can be noticed that the last 3 diseases have been shown to be linked to OA. Recently, Weber et al. [42] demonstrated an association between OA and the risk of dementia. The miR-186 belonged to a set of 27 differentially expressed miRNAs between patients suffering from Alzheimer’s disease and controls [43]. The pathways potentially downregulated are linked with neuronal synaptic functions. Moreover, neuropathic pain is at least partially responsible for pain in OA [44]. Interestingly, miR-186 negatively regulated CXCL13, also known as B lymphocyte chemoattractant, and its downregulation in spinal neurons after spinal nerve ligation causes upregulation of CLCX13 to drive neuropathic pain [45]. Finally, several cross-sectional studies have shown a strong association between OA and cardiovascular diseases [46], and the overexpression of miR-186 in plasma have the potential to be used for the early detection of myocardial infarction [47]. However, for the present analysis, no woman suffered from dementia. Moreover, we had incomplete information regarding cardiovascular events, and we did not have data concerning pain in the OFELY study. So, the potential links between miR-186-5p, cardiovascular diseases, dementia, and OA could not be evaluated.

 Our study has strengths and limitations. We investigated the association between the serum miRNAs expression and prevalence and for the first time incidence of knee OA in a well-characterized population of women followed prospectively over a long period of time of 4 years. We have chosen to carry out the validation step of our study by analysis of duplicate samples on TaqMan microRNA arrays, in order to reduce erratic manipulations and improve the reproducibility between the samples. Moreover, our study combines the validation of our NGS results and the first attempt to replicate previously published results, a process that had been lacking in the miRNA research field. Finally, we have indicated that women selected for the analyses of prevalent and incident knee OA had also OA at other sites (Table 1). This is a key point because we showed in the OFELY cohort that at this mean age, 68 ± 8 years, the prevalence of OA was high with 75% and 88% of women having osteophytosis at the lumbar spine and thoracic spine, respectively [48, 49] in agreement with previous prevalence data [50, 51]. Consequently, in both groups, knee OA and healthy women, we have verified that the presence of OA at other sites did not cause any bias between the groups (see Table 1) in order to decrease as much as possible the effects of this unavoidable situation of generalized OA on the detection of miRNAs associated with knee OA. However, we studied knee OA in women only, and the number of participants is limited. It has been demonstrated that gender may influence the level of the expression of miRNAs [52]. Consequently, our results need to be confirmed by additional larger studies and particularly in males. It can be noticed that miR-146 and miR-186 have been quantified in serum patients because the miR-3p forms of both miRNAs were almost undetectable in the 20 tested sera in the screening phase (data not shown). This lack of detection in the serum is probably related to thermodynamic instability of the 3p arms and to their subsequent degradation in the cellular p-bodies. Finally, the molecular mechanisms associating serum miR-146 and miR-186 to OA remain to be further clarified, for example, the role of the single-nucleotide polymorphisms in the promoter region of miR-146 gene [53].

Collectively, our results show that miR-146a-5p is increased in women with mild to moderate knee OA compared to healthy women. Importantly, miR-186-5p is also increased in those women who will develop radiographic knee OA over the next 4 years; therefore, this miRNA has the potential to detect preclinical knee OA.

## Supplementary information

**Additional file 1: Data S1.** Protocol details for NGS analysis, RNA isolation and RT-qPCR analysis.

**Additional file 2: Data S2.** Mean and standard deviation (SD) for each quartile of the 19 miRs tested in the validation phase.

## Data Availability

All data generated and analyzed during this study are included in this published article.
