# Supplementary material for: Association of circulating microRNAs with prevalent and incident knee osteoarthritis in women: the OFELY study
Source: Arthritis Res Ther. 2020 Jan 2;22:2. doi: 10.1186/s13075-019-2086-5 (PMC6941326; doi:10.1186/s13075-019-2086-5)
Supplement: Supplementary file 1 — Additional file 1: Data S1. Protocol details for NGS analysis, RNA isolation and RT-qPCR analysis. [file 13075_2019_2086_MOESM1_ESM.docx]

**Supplementary data 1**

**NGS analysis**

According to the manufacturer’s protocol (EXIQON, Denmark), total RNA extraction was performed from 400 µl of serum followed by the miRNA sequencing (Illumina platform). Total RNA (6 μl) extracted from serum with the miRCURY Biofluids extraction kit (Exiqon®, Danemark) was converted into miRNA NGS libraries using NEBNEXT library generation kit (New England Biolabs Inc.) according to the manufacturer’s instructions. Adaptaters containing Unique Molecular Index (UMI) were ligated into 3’-OH and 5’-P prior to the reverse-transcriptase reaction to generate cDNA. The cDNA was pre-amplified with a 18 cycle PCR with primers containing sample specific indexes. The libraries were purified on QiaQuick columns and the insert efficiency evaluated by Bioanalyzer 2100 instrument on high sensitivity DNA chip (Agilent Inc.) The miRNA-cDNA libraries were size fractionated on a LabChip XT (Caliper Inc.) to excise the bands representing adaptors and 15-40 bp insert. Samples were then quantified by qPCR and the libraries were pooled in equimolar concentrations. After a final quantification by qPCR, optimal concentration of the library pools was used to generate the clusters on the surface of a flowcell before sequencing using v3 sequencing methodology according to the manufacturer instructions (Illumina Inc.).

Following the sequencing on a NextSeq500 sequencing instrument (Illumina), the intensity correction and base calling, the data was quality checked by assigning Q-scores. A Q-score above 30, indicating an error probability for incorrect base call of 0.001, assessed for high average read quality of the UMI-corrected reads. After de-multiplexing of raw data and correction for amplification biais, reads were aligned to miRNA sequences known in miRBase20.

**RNA extraction**

Total RNA was extracted from 200µl serum with the miRCURY Biofluids extraction kit) according to manufacturer recommendations (Exiqon®, Danemark). Samples were thawed on ice and centrifuged at 3,000g for 5 minutes. A lysis buffer solution containing 1µl of glycogen as RNA carrier and a synthetic spike-in control RNA (cel-miR-39-3p) was added to the serum. After cellular lysis and protein precipitation, supernatant was placed on a silica micro-column, treated with rDNAse, washed first with a buffer to inhibit rDNAse and then with a solution containing ethanol. RNA was eluted with 40 µl RNAse/DNAse-free water (Invitrogen®) and stored at -80°C.

**miRNA real-time qPCR analysis**

MiRNAs were quantified by TaqMan® Advanced miRNA technology (Applied Biosystems, ThermoFisher Scientific) in which cDNA was prepared from 2μl of total RNA using a TaqMan Advanced miRNA cDNA synthesis kit. Briefly, the kit uses 3' poly-A tailing and 5' ligation of an adaptor sequence for each end extension of the mature miRNAs present in the sample, prior to reverse transcription (RT). Universal RT primers anneal to the universal sequences present on both the 5' and 3' extended ends of the mature miRNAs. Quantification of miRNAs expressed at low copy number was improved by cDNA amplification in a 2X TaqMan PreAmp master mix containing Megaplex PreAmp primers.

The qPCR amplification was performed on 1:10 dilution of cDNA obtained by miRNA-Amp reaction on 5 µl of the RT reaction, using the 2X Fast Advanced Master Mix and the 20X TaqMan® Advanced miRNA Assays, placed in the wells of pre-designed miRNA TaqMan arrays (Applied Biosystems). These TaqMan ®Advanced miRNA assays contain pre-formulated primers and TaqMan ®MGB (minor groove binder) probes that allow the recording of fluorescence signal in the PCR reaction (Table 3). Amplified cDNAs (15µl) were mixed with 75 µl of TaqMan Fast Advanced Mastermix in H_2_0 (60µl) and 100 µl of each sample were added to the array tanks of the miRNA TaqMan arrays. These cards were centrifuged (Thermo scientific) to distribute the reaction medium in the wells, sealed (Applied) according the manufactured protocol, prior to analysis. The TaqMan array microRNA cards were designed for the quantification in duplicate of 19 miRNAs by RT-qPCR reaction on a QuantStudio® 7 flex (Applied Biosystems) according to the manufacturer’s protocol. The C_T_ (threshold cycle value) was recorded as the cycle number at which the fluorescence generated within a reaction crosses the fluorescence threshold, a fluorescent signal significantly above the ROX™ fluorescence background recorded in each sample. We used the software Expression Suite (Applied Biosystems) to express the miRNA level as relative quantification (RQ). The Ct values of each miRNA were normalized with the mean of expression level of three endogenous miRNAs (miR-191-5p, miR-222-3p and miR-361-5p) that are ubiquitously expressed and unrelated to the metabolism of bone and joint tissues. RQ was calculated as 2^–ΔΔCT^, with ΔC_T_ = (C_T_ miRNA – C_T_ mean of the 3 endogenous controls) and ΔΔC_T_ = (ΔC_T_ of the miRNA – ΔC_T_ mean of the miRNA through all samples) and converted as Fold Change (FC) = Log_2_(2^–ΔΔCT^) (28). The exogenous spike cel-miR-39-3p was used as a qPCR quality control (Table 3).
