# Supplementary material for: Association of circulating microRNAs with prevalent and incident knee osteoarthritis in women: the OFELY study
Source: Arthritis Res Ther. 2020 Jan 2;22:2. doi: 10.1186/s13075-019-2086-5 (PMC6941326; doi:10.1186/s13075-019-2086-5)
Supplement: Supplementary file 2 — Additional file 2: Data S2. Mean and standard deviation (SD) for each quartile of the 19 miRs tested in the validation phase. [file 13075_2019_2086_MOESM2_ESM.pptx]

## Slide 1
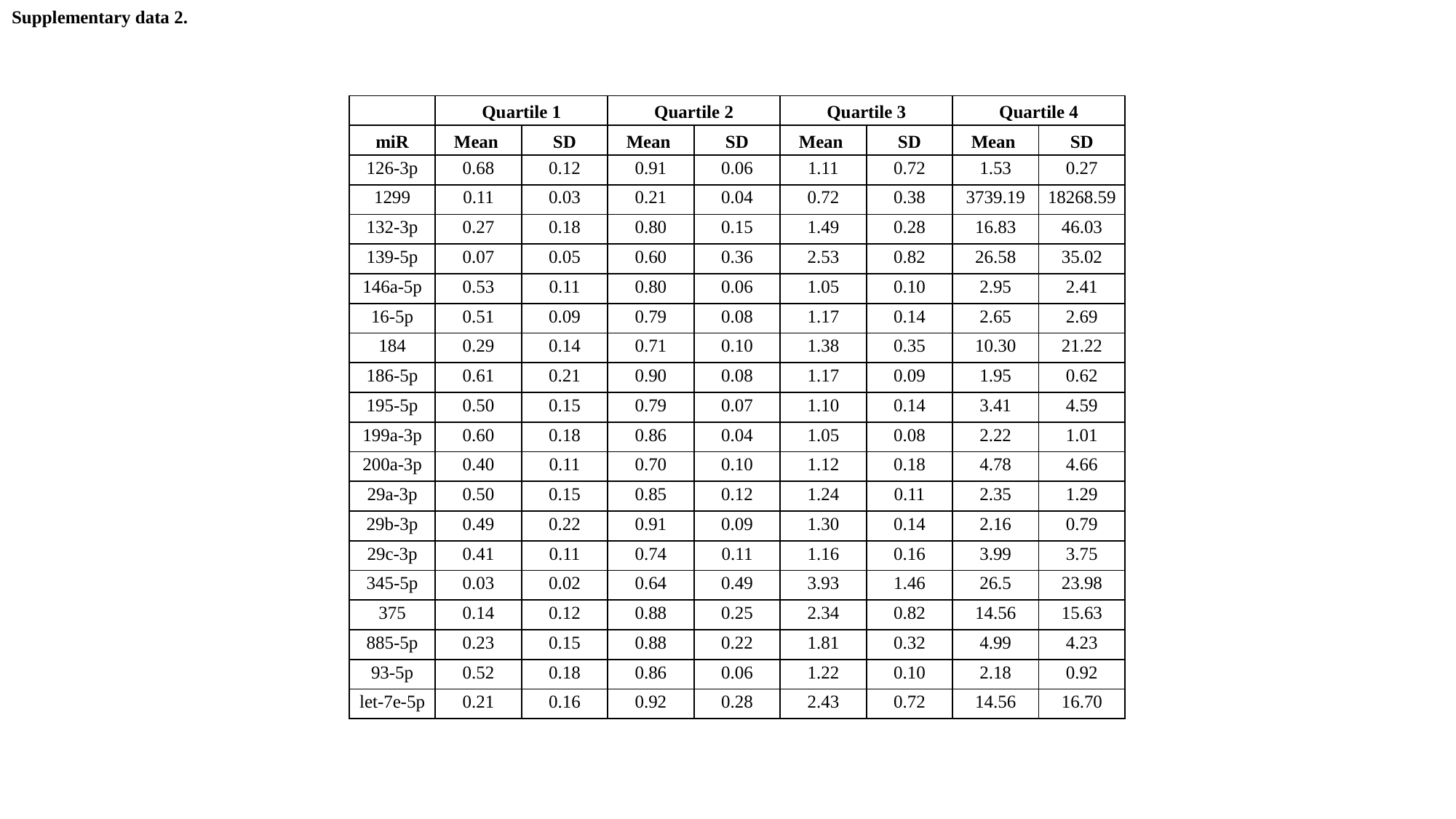

Supplementary data 2.
| | Quartile 1 | | Quartile 2 | | Quartile 3 | | Quartile 4 | |
| --- | --- | --- | --- | --- | --- | --- | --- | --- |
| miR | Mean | SD | Mean | SD | Mean | SD | Mean | SD |
| 126-3p | 0.68 | 0.12 | 0.91 | 0.06 | 1.11 | 0.72 | 1.53 | 0.27 |
| 1299 | 0.11 | 0.03 | 0.21 | 0.04 | 0.72 | 0.38 | 3739.19 | 18268.59 |
| 132-3p | 0.27 | 0.18 | 0.80 | 0.15 | 1.49 | 0.28 | 16.83 | 46.03 |
| 139-5p | 0.07 | 0.05 | 0.60 | 0.36 | 2.53 | 0.82 | 26.58 | 35.02 |
| 146a-5p | 0.53 | 0.11 | 0.80 | 0.06 | 1.05 | 0.10 | 2.95 | 2.41 |
| 16-5p | 0.51 | 0.09 | 0.79 | 0.08 | 1.17 | 0.14 | 2.65 | 2.69 |
| 184 | 0.29 | 0.14 | 0.71 | 0.10 | 1.38 | 0.35 | 10.30 | 21.22 |
| 186-5p | 0.61 | 0.21 | 0.90 | 0.08 | 1.17 | 0.09 | 1.95 | 0.62 |
| 195-5p | 0.50 | 0.15 | 0.79 | 0.07 | 1.10 | 0.14 | 3.41 | 4.59 |
| 199a-3p | 0.60 | 0.18 | 0.86 | 0.04 | 1.05 | 0.08 | 2.22 | 1.01 |
| 200a-3p | 0.40 | 0.11 | 0.70 | 0.10 | 1.12 | 0.18 | 4.78 | 4.66 |
| 29a-3p | 0.50 | 0.15 | 0.85 | 0.12 | 1.24 | 0.11 | 2.35 | 1.29 |
| 29b-3p | 0.49 | 0.22 | 0.91 | 0.09 | 1.30 | 0.14 | 2.16 | 0.79 |
| 29c-3p | 0.41 | 0.11 | 0.74 | 0.11 | 1.16 | 0.16 | 3.99 | 3.75 |
| 345-5p | 0.03 | 0.02 | 0.64 | 0.49 | 3.93 | 1.46 | 26.5 | 23.98 |
| 375 | 0.14 | 0.12 | 0.88 | 0.25 | 2.34 | 0.82 | 14.56 | 15.63 |
| 885-5p | 0.23 | 0.15 | 0.88 | 0.22 | 1.81 | 0.32 | 4.99 | 4.23 |
| 93-5p | 0.52 | 0.18 | 0.86 | 0.06 | 1.22 | 0.10 | 2.18 | 0.92 |
| let-7e-5p | 0.21 | 0.16 | 0.92 | 0.28 | 2.43 | 0.72 | 14.56 | 16.70 |
